# Development and Pilot Testing of 24-Hour Multiple-Pass Recall to Assess Dietary Intake of Toddlers of Somali- and Iraqi-Born Mothers Living in Norway

**DOI:** 10.3390/nu6062333

**Published:** 2014-06-19

**Authors:** Navnit Kaur Grewal, Annhild Mosdøl, Marte Bergsund Aunan, Carina Monsen, Liv Elin Torheim

**Affiliations:** 1 Fafo Institute for Applied International Studies, P.O. Box 2947 Tøyen, Oslo NO-0608, Norway; E-Mail: liv.elin.torheim@hioa.no; 2 Department of Health, Nutrition and Management, Faculty of Health Sciences, Oslo and Akershus University College of Applied Sciences, P.O. Box 4 St. Olavs plass, Oslo NO-0130, Norway; E-Mails: annhild.mosdol@kunnskapssenteret.no (A.M.); marteaunan@hotmail.com (M.B.A.); carina_monsen86@hotmail.com (C.M.); 3 Department of Evidence Summaries, The Norwegian Knowledge Centre for Health Services, P.O. Box 7004 St. Olavs plass, Oslo NO-0130, Norway

**Keywords:** 24-h recall, dietary assessment, infants, toddlers, Somalia, Iraq, immigrants, Norway

## Abstract

The aim of this study was to develop, test, and evaluate a 24-h recall procedure to assess the dietary intake of toddlers of Somali- and Iraqi-born mothers living in Norway. A protocol for a 24-h multiple-pass recall procedure, registration forms, and visual tools (a picture library for food identification and portion size estimation) was developed and tested in 12 mothers from Somalia and Iraq with children aged 10–21 months. Five female field workers were recruited and trained to conduct the interviews. Evaluation data for the 24-h recall procedure were collected from both the mothers and the field workers. Nutrient intake was calculated using a Norwegian dietary calculation system. Each child’s estimated energy intake was compared with its estimated energy requirement. Both the mothers and the field workers found the method feasible and the visual tools useful. The estimated energy intake corresponded well with the estimated energy requirement for most of the children (within mean ± 2 SD, except for three). The pilot study identified the need for additional foods in the picture library and some crucial aspects in training and supervising the field workers to reduce sources of error in the data collection.

## 1. Introduction

Dietary assessment studies are important for the development of public nutrition policies and interventions because they can identify population groups at risk of nutritional health problems and describe their dietary habits [1]. In Europe, some immigrant groups have a higher risk of developing nutrition-related diseases than the host population, in particular overweight/obesity and diabetes mellitus type 2 [2,3,4]. This may be due to changes in dietary habits and physical activity patterns influenced by a process of acculturation, urbanization and westernization [2,5]. Only relatively few studies describe dietary habits in immigrant groups, but these indicate diets with increased consumption of processed food after migration that replace healthy dietary components, such as fruits, vegetables, nuts, and whole grains [2,3].

Dietary studies among immigrant groups are hampered by a lack of suitable cultural-sensitive assessment methods and data collection procedures. Missing food composition data on ethnic foods and the use of dietary assessment methods, which are not critically assessed for suitability in these groups, may limit the reliability of dietary intake data among immigrants [2]. Furthermore, various methodological aspects, such as sampling and recruitment, tools and method of administration, among others, often require special attention [6]. In addition, dietary assessment among infants and children in particular has several inherent challenges, and these might be amplified among immigrant groups [1,7]. It is, therefore, important to exercise considerable caution when conducting dietary studies in this study group in order to reduce possible errors and increase validity.

Previous Norwegian national dietary surveys among infants (“Spedkost”, aged six months and 12 months) and toddlers (“Småbarnskost”, aged 24 months) excluded children of mothers born outside Scandinavia [8,9,10]. There are two main reasons for this exclusion: (1) the dietary assessment method used in the study was a food frequency questionnaire (FFQ) and the food list was not adapted to non-Scandinavian food habits and (2) the FFQ was only available in Norwegian. Thus, the method was less suitable for population groups with atypical food habits, poor Norwegian skills or lower literacy levels. The researchers expressed a need for separate studies among children of immigrant parents using more appropriate methods [11].

The “InnBaKost” study was initiated to address the limited knowledge about dietary habits and health among children in Norway with immigrant backgrounds. Children of Somali- and Iraqi-born mothers were chosen because they are the two non-Western immigrant groups currently with the highest number of births in Norway [12]. The aim of this research project was to collect information about breastfeeding practices and feeding patterns among infants aged six months with follow-up at 12 and 24 months to supplement the Spedkost and Småbarnskost surveys [8,9,10]. A modified FFQ was used for the data collection at six months of age. However, a structured 24-h recall method was considered more appropriate at ages 12 and 24 months. This because the FFQ requires that variations in food habits must be known and included in the food list to develop suitable FFQs [13,14]. The aim of the present study was to develop, pilot test and evaluate a protocol for a 24-h recall procedure, with registration forms and visual tools, to assess the dietary intake of toddlers of Somali- and Iraqi-born mothers living in Norway.

## 2. Methods

### 2.1. Subjects and Study Design

The pilot study was carried out January–June, 2013. Twelve Somali- and Iraqi-born mothers with children aged 10–21 months living in Oslo and Akershus counties, Norway, were recruited through several methods: the Norwegian National Population Register, open kindergartens and by using the snowball method. Inclusion criteria were the mothers’ country of birth and the child being approximately 12 months old, born in Norway and with no serious health problem or disease requiring a special diet. The mothers received a bilingual information letter and provided written consent. Respondents received a shop voucher after completing two recalls. The study was approved by the Norwegian Regional Committees for Medical and Health Research Ethics.

To measure the dietary intake among toddlers, a structured 24-h recall method was used [15]. In the 24-h recall method, the mothers were interviewed twice, usually 1–2 weeks apart, by trained field workers about the exact food and beverage intake of their child during the preceding 24 h. If other caretakers were involved, the mother was asked to obtain information about the child’s food consumption while under their care. A researcher (C.M., M.B.A., or N.K.G.) was present and observed the interviews. In addition to the food and beverage intake of the child, information about the performance of the interviews and methods was collected through an evaluation form.

### 2.2. The 24-H Recall Method

#### 2.2.1. Picture Library for Food Identification

To help the mothers and the field workers identify the correct foods given to the child, a library with pictures of food items commonly eaten by children in Norway was developed. A list of food items to be included in the library was made based on knowledge about Norwegian children’s dietary intake. In addition, food items identified as eaten by Somali and Iraqi children through an informal qualitative prestudy and food items suggested by the field workers were also included. The foods were photographed in supermarkets and independent shops owned by immigrants in Oslo, with permission from the owners/managers. A Canon Ixus 860 IS digital camera was used, and the pictures were edited in iPhoto on a MacBook Pro (Apple Inc., Cupertino, CA, USA).

The library contained pictures of a wide selection of industrially produced baby foods for children aged 8–15 months, as well as other foods and beverages. Before the pilot study, the field workers suggested adding Nido milk powder, different types of meat, cheese and biscuits. The “bread scale”, a Norwegian labeling scheme for fiber and wholemeal content of bread, was also included in the library.

To ease retrieval of pictures during the interviews, the 336 unique pictures were categorized in 19 different folders on an iPad (Table 1). The folders contained between 4 and 50 pictures, with the largest number of pictures in the folder “fruits and vegetables”. Because certain foods may be categorized differently by different people, some pictures were placed in more than one folder. For instance, smoothies appeared both in the folders “snacks” and “juice and nectar”. Thus, the final library contained 405 pictures in 19 folders.

**Table 1:** Number of pictures in each folder of the picture library.

Food Folder	Number of Pictures
Baby cereals	16
Snacks	33
Infant formula	16
Ready-made meals	10
Bread spreads	19
Dinner	39
Yoghurt and desserts	29
Oils and butter	13
Dairy products	13
Fruits and vegetables	50
Breads	22
Pasta, rice and beans	17
Supplements	8
Milk	38
Juice and nectar	46
Soda	4
Squash, lemonade, *etc.*	17
Meat	4
Biscuits	11
**Total**	**405**

#### 2.2.2. Photographic Booklet and Measuring Equipment for Portion Size Estimation

This study used a photographic booklet for portion size estimation developed for the Spedkost and Småbarnskost surveys [8,9,10]. It included 17 color photograph series of selected food items representing different, usually four, portion sizes appropriate for toddlers ranging from small (A) to large (D), with up to six different portion sizes for baby cereal. The mother used the booklet as a tool to identify portion sizes eaten by her child. The field workers also brought a kitchen scale and three measuring cups (in deciliters and milliliters) to weigh or measure foods or volume in tableware from the respondents’ homes whenever possible. Frequently, both methods were used to compare the results.

#### 2.2.3. 24-H Recall Protocol and Registration Form

The protocol contained instructions on how the field workers should conduct the 24-h recalls based on standard procedures for face-to-face 24-h recall in the literature [16]. A 24-h period was defined as starting at the time the child woke up the previous day until the time the child woke up the day of interview. The field worker informed the mother about the recall procedure at the beginning of each interview and recorded the answers in specially designed paper-based forms that matched the three-stage, multiple-pass interviewing technique [16]. In the first pass, the mother was asked to give a complete overview of all foods and beverages consumed during the 24-h period. If the mother was still breastfeeding her child, each breastfeeding occurrence was registered. In the second pass, a detailed description of each food and beverage consumed were obtained. This included type of product, brand names, cooking methods, amounts, and food leftovers. In this pass, the picture library was used to identify foods, and the photographic booklet and measuring equipment to estimate amounts. The field workers used standardized probe questions to collect specific details. In the last pass, the field workers summarized and reviewed the information to ensure that all items were recorded correctly. This phase also included a checklist of foods and beverages that are often forgotten, such as water, snacks, and supplements. Representativeness of the day and food allergies/intolerances was also registered. A separate questionnaire covered background information of the mother and child.

### 2.3. Training of Field Workers

Five female field workers were recruited to conduct the interviews, of which two spoke Somali and three Arabic. All of them spoke fluent Norwegian and one of the Arabic-speaking field workers also spoke Kurdish. Thus, the mothers could choose to speak either Norwegian or their own language during the interviews. 

The field workers received 1–2 weeks of training on how to conduct 24-h recalls according to the protocol using the forms and tools. Practice took place in pairs and in plenary using different languages. The training particularly emphasized how to ask follow-up questions to make sure all food items were registered, to identify the correct food items and to estimate portion sizes as accurately as possible.

### 2.4. Pilot Testing of the Procedures for 24-H Recall

The pilot study enabled a full appraisal of all aspects of the 24-h recall procedure. The field worker and the observer recorded data and answered questions regarding the method after each interview using an evaluation form (Table 2). The mothers were asked for their views on the method, including the visual tools, after the second interview.

The dietary data obtained from the 24-h recalls were manually coded and entered by C.M., M.B.A., and N.K.G. in a software system (KBS, database AE-10) developed at the Department of Nutrition, University of Oslo, Norway. The food database in KBS was mainly based on the official Norwegian food composition table. Breast milk intake among the breastfed children was calculated by multiplying the number of feeding events by an estimated breast milk intake per feed of 124 mL. This amount of breast milk per feed was derived from an estimated daily breast milk intake of 497 mL among 12-month old children in developed countries [17] divided by the average breastfeeding frequency in Norwegian 12-month old breastfed children of 4 times per day [9] (497 mL/4 feeds = 124 mL/feed).

As an objective measure of validity for this pilot test, each child’s estimated energy intake (EEI) was compared with its estimated energy requirement (EER). According to the Nordic Nutrition Recommendations, estimated average daily energy requirement for 12-month-old boys is 337 kJ/kg and 333 kJ/kg for girls [18]. We did not measure the children’s weight, but recorded the weight registered at clinic for the 12-month health check-up (Table 3). However, eight of the children were interviewed at ages between 13 and 21 months and two were interviewed younger than 12 months of age. One of the children younger than 12 months of age had his body weight measured at the 8-month consultation at the child health center. Thus, for 9 of the 11 children with registered body weight, at least one month had passed between the weighing and the 24-h recall. To adjust for this, an estimate of monthly weight gain was calculated using the World Health Organization’s growth standards for children between 0 and 24 months [19]. The estimated weight gain for boys 8–21 months of age varies by month and is highest from 8 to 9 months (3.25%) and decreases gradually to 1.76% from 20 to 21 months. For girls, weight gain from 12 to 13 months was found to be 2.64% and from 13 to 14 months average weight gain is 2.46%. Each child’s body weight was thus calculated by adding the monthly estimated weight gain to its weight. For the child with no records of body weight registered, the average weight of 11-month old girls was used. Using the estimated body weight at the time of interview, EER for each child was calculated and compared with the EEI calculated from the 24-h recalls, using the mean intake of the two recalls. Differences between EER and EEI were tested with paired samples *t*-test. Bland-Altman plot [20] was used to visualize the dispersion between EER and EEI. Linear regression analysis was applied to study whether there was any relationship between the mean of the estimates EER and EEI and the difference between the two estimates.

**Table 2:** Evaluation form for the pilot study.

Source of Information	Evaluation Topic	Question Asked
Observation by researchers	Time spent by the field worker	Time spent on picture library (iPad)? Time spent on photographic booklet? Time spent on measuring equipment? Other notes?
	Use of visual tools	Which pictures were used most frequently or not at all?
	Standardisation of methods/field workers	Did the field workers ask the questions in the same way? Did they follow the protocol? Did they use the visual tools?
Questions to respondents	Clarity of questions	Were any of the questions difficult to answer/unclear? If yes, which and why?
	Missing pictures	Did you miss pictures of any foods/beverages? Are there some foods/beverages you give your child often, but not yesterday? Are there any other foods/beverages you know Somali/Iraqi children often eat/drink?
	Portion sizes	Did the portion sizes in the booklet match the portion sizes your child usually eats? Was it easier to estimate the amount the child had eaten by using the booklet, measuring equipment or by showing it on/in the plate/cup used?
Questions to field workers	24-h recall protocol	Was the protocol easy to understand? If no, why not? How did you experience the different passes during the interview? Was it easy to distinguish these from each other?
	Picture library	How did you experience using the picture library during the interview? Was it user friendly? If no, why not?
	Photographic booklet	How did you experience using the photographic booklet to estimate portion sizes? Did you miss photos of any foods/beverages?
	Measuring equipment	How did you experience to estimate amounts using the measuring equipment? Did you miss any equipment?
	Registration form for 24-h recall	How did you experience using the form? Was the order of items logical to you? Was there enough space to write? If no, where did you want more space?

**Table 3:** Estimated energy requirements and energy intake among children (10–21 months) with mothers from Iraq (ID 1–5) and Somalia (ID 6–12) living in Norway.

ID	Sex	Estimated Average Daily Energy Requirements (kJ/kg)	Body Weight at 12 Months (g)	Age at Time of Interview (months)	Estimated Body Weight at Time of Interview (g) ^a^	EER at Time of Interview (kJ/day) ^b^	EEI at Time of Interview (kJ/day) ^c^	Percentage Differences between EER and EEI (%) ^d^
**1**	F	333	8440	14	8855	2949	2843	−4
**2**	M	337	11,600	13	11,864	3998	3486	−14
**3**	M	337	11,083	21	13,268	4471	3649	−20
**4**	F	333	8719 ^e^	11	8719	2903	4415	41
**5**	M	337	8300	12	8300	2797	3102	10
**6**	F	333	10,000	13	10,246	3412	4043	17
**7**	M	337	8200	12	8200	2763	2764	0
**8**	F	333	9970	14	10,460	3483	3157	−10
**9**	M	337	10,000	14	10,466	3527	2232	−45
**10**	M	337	11,000	14	11,513	3880	4635	18
**11**	M	337	9890 ^f^	10	10,509	3542	3556	0
**12**	F	333	9270	13	9498	3163	4636	38
**Mean**		**335**	**9706**	**13**	**10,158**	**3407**	**3543**	**18^g^**
**SD**		**2**	**1146**	**3**	**1544**	**527**	**773**	**15^g^**

^a^ Estimated body weight at time of interview calculated based on average growth rate from World Health Organization’s growth standards [19] multiplied by number of months between time of weighing and time of interview; ^b^ Estimated body weight at time of interview multiplied with estimated average requirement per kilogram; ^c^ Mean estimated energy intake of the two recalls; ^d^ Calculated as percent difference of mean. Difference between EER and EEI tested with paired samples *t*-test: *p* = 0.58; ^e^ Body weight not registered. Average weight for girls at 11 months of age used as reference [19]; ^f^ Body weight at 8 months of age; ^g^ Calculated using absolute values of percentage differences

## 3. Results

### 3.1. Subjects

A total of 28 Somali-born mothers were asked to participate in the pilot study, and 13 consented. However, only seven of these showed up to the appointed interview. Of the fourteen 24-h recalls, eight were conducted in Norwegian and six in Somali. Likewise, 48 Iraqi mothers were contacted, seven consented, but only five showed up at the interview. Five of the 24-h recalls were conducted in Kurdish, four in Arabic and one in Norwegian. Among the 12 participating mothers, mean age was 31 (range 22–42) and the average number of years lived in Norway was 15 (range 3–24). Three mothers had no education from Norway. Two of them had, however, completed a Norwegian course. Seven mothers had completed high school education and two had completed higher education. Seven mothers had more than one child.

### 3.2. Results from the Evaluation Form

The mean (minimum-maximum) time spent on the total 24-h recall interviews was 47 (20–75) min. There was a decreasing trend in the time spent on each interview conducted over time in both the Somali and Iraqi groups. The repeat interviews were conducted by the same field worker, except for three of the Somali mothers and one Iraqi mother where two different field workers conducted the interviews.

The field workers spent an average of four minutes during each interview showing pictures on the iPad. The visual tools were used during all interviews. Of the 19 folders in the picture library, eight were used by the Somali mothers to identify foods. The most frequently used folders were “baby cereal” (seven interviews) and “oils and butter” (four interviews). Among the Iraqi mothers, pictures from 13 of the 19 folders were used. The most frequently used folders in this group were “breads” (eight interviews) and “baby cereal” (four interviews). Although the mothers browsed through all folders to identify foods given to the child, none of the Somali or Iraqi mothers used or identified foods from the five folders “ready-made meals”, “fruits and vegetables”, “soda”, “squash, lemonade, *etc.*” or “meat”.

Eleven of the 17 colored photograph series were used by the Somali mothers to estimate portion sizes eaten by the child. The portion sizes of baby cereal and butter were the most frequently used total in 10 and 9 of the 14 interviews, respectively. The Iraqi mothers used 10 of the 17 photograph series of portion sizes to estimate foods consumed by the child. The most frequently used series were the portion sizes for milk and butter, which were referred to in 9 and 5 of the 10 interviews, respectively. The measuring equipment was used together with the photographic booklet in the first interviews, but over time the interviewers favored the photographic booklet over actual measurements. Reasons given for this shift were that measurements were time consuming and difficult to use when the interviews were conducted outside the informants’ homes. When mothers were asked to identify amount with both the photographic booklet and by measurements of actual foods, these seemed to correspond well.

The protocol was mostly only used during the last pass, when the field workers were going through the checklist of foods and beverages often forgotten. When the interviews were conducted in Norwegian, the observers noted that the field workers consistently asked about added foods/ingredients, brands and amounts consumed. It was sometimes difficult for the field workers to write down recipes and cooking methods because of limited space on the forms. However, the amount of food eaten by the child was usually asked about and written down clearly.

The mothers expressed that the picture library was a good tool to be reminded of and to identify the type of foods given to the child. It was especially useful for remembering brand names. Among pictures missing in the picture library, some mothers mentioned different types of rice, fruit purees, bread spreads, breads, butter, baby cereals, and yoghurts, as well as Weetabix and prunes.

One of the topics that emerged repeatedly was how difficult it was to estimate portion sizes. Six mothers mentioned the difficulties in estimating the amount of bread eaten by the child, without pictures of bread in the booklet. Other pictures of portion sizes mentioned as missing were lasagna, spaghetti and pancakes. Five mothers expressed that pre-packed industrially produced foods were easier to estimate. All mothers found that the portion size options in the photographic booklet matched amounts the child usually ate. Ten mentioned that it was easier to show amounts of foods eaten using the booklet, whereas illustrating amounts of beverages was easier using the measuring equipment.

Most mothers said that the day of interview represented the typical foods given to the child, only three recall days were considered non-representative for the typical foods given. Two foods (bread and bulgur) were mentioned by two mothers as being typical foods given to the child, but not during the days in question. Pancakes (*anjera*), Weetabix and juice were mentioned by two Somali mothers as cultural relevant foods often given to children, while four Iraqi mothers mentioned different types of staple foods and vegetables, such as okra.

All field workers found the protocol easy to understand. Finding pictures in the picture library took time to begin with, but became easier after a few interviews. The photographic booklet was judged as a good tool for estimating portion sizes. However, similarly to the mothers, they missed portion size pictures of pasta and bread.

The registration form was described as clear and easy to understand, but the field workers missed more space to write down recipes.

### 3.3. Results from the 24-H Recalls

Six mothers were still breastfeeding their children (four Iraqi and two Somali mothers). The average breastfeeding frequency was two times per day (range one to four times per day). Foods given to the children included bread, porridge, different fruit and vegetables, snacks and supplements. The porridge was either industrial produced or home-made using oatmeal or bulgur. A couple of mothers also added margarine, olive oil, salt and/or sugar when preparing the porridge. Some of the foods registered that are not commonly given to Norwegian children were nan bread, feta cheese, Turkish delight, bulgur porridge, pancakes (*anjera*), and seeds. None of the mothers reported using ready-made dinners, as most of them mentioned that they did not trust the contents and the ready-made dinners were not considered to be fresh. The home-made dinners often constituted of different staple foods, vegetables, meat, and fish.

### 3.4. Results from Energy Intake Estimation

**Figure 1:**
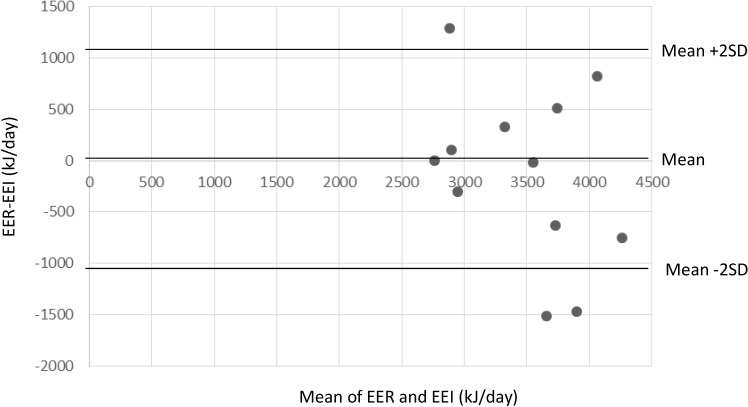
The difference between estimated energy requirement (EER) and estimated energy intake (EEI), plotted against the mean of EER and EEI (*n* = 12). SD = Standard deviation.

Table 3 presents the comparison of each child’s EER with its EEI calculated from the 24-h recall. The percent difference between EER and EEI was in the range of ±0%–10% for five of the children and in the range of ±11%–20% for four of the children, whereas for three of the children the percentage of difference between the two estimates was ±38%, 41% or 45%, respectively. The mean (SD) for EER and EEI was 3407 (527) kJ/day and 3543 (773) kJ/day, respectively, and the difference was not significant, as tested with paired samples *t*-test. The Bland-Altman plot (Figure 1) showed large individual variations in the differences between EER and EEI but no clear pattern. A linear regression analysis testing the relationship between the mean of the estimates EER and EEI and the difference between the two estimates was not significant, *p* = 0.24. This indicates that the difference between the two estimates is not related to the magnitude of the estimates. Excluding child number 4, for whom there was no registered weight, did not change the results.

## 4. Discussion

For the InnBaKost study, we developed a protocol for a 24-h recall procedure, including a picture library to assist in identifying the correct foods eaten. In addition, a photographic booklet was used for portion size estimation. Although the latter approach has become a common method for portion size estimation [21,22], including dietary assessment in children [23,24], the use of a picture library is a rather novel approach. The hypothesis was that the picture library would be a useful tool to identify the correct food and brand, particularly for dietary assessment among immigrant mothers with varying levels of language and literacy skills.

A review conducted by Burrows *et al.* (2010), indicates that weighed food records provide the best dietary estimates for younger children aged 0.5 to 4 years, while 24-h multiple-pass recall that uses parents as reporters is the most accurate method to estimate total energy intake in children aged 4 to 11 years [25]. The weighed food record method requires both motivation and good literacy skills and is often time-consuming. Thus, the method has been considered to be less suitable for dietary assessment in immigrants, as the method has led to misreporting and dropout in immigrant groups due to the burden and time consumption the method carries [26]. The face-to-face FFQs and multiple-pass 24-h recalls are reported to be the two most frequently used methods with immigrant populations in Europe [6]. The 24-h recall is more flexible because it can capture all foods and beverages consumed the preceding day, with no assumptions about the food culture or dependency on literacy levels. In addition, as seen from the few recalls in this pilot, some mothers gave selected atypical foods to their children and mostly made home-made dinners, which may vary from the general Norwegian population in regards to composition and preparation method. The 24-h recall has therefore been recommended as the most optimal method for many immigrant groups and is considered to provide valid information among children [26,27]. In addition, the interactive nature and the personal contact of the method may contribute to more reliable data collection, although social desirability bias may cause some misreporting [28]. The multiple-pass technique is considered to give the most exact estimates, and limit misreporting, because the probing questions encourage the respondent to remember more of the foods consumed [16]. The respondent burden is usually small compared to weighed records [29].

The protocol was used sparingly during the interviews, because the field workers expressed that they already knew the content in the protocol and that it was difficult to focus on the protocol while registering the child’s food consumption. Thus, it was recommended that important guidelines from the protocol could be included in the 24-h recall registration form instead. The decreasing time spent on the second interview with each mother was mostly due to the mother being more prepared and that the background information was already collected. Another reason for the decline in time spent may have been that the field workers became more familiar with the method and navigated the picture library and photographic booklet more easily. The measuring equipment was initially used together with the photographic booklet to see how well both measurements corresponded, but both the field workers and mothers expressed that it was too time-consuming.

Both the mothers and field workers reported the picture library to be a good tool to identify foods given to the child. It was mostly used when the interviews were conducted outside the respondents’ homes because the mothers could show foods available in the home. The use of a picture library similar to this has not been described by many; however, the use of photo images has been reported to be useful as a memory aid for respondents during 24-h recalls [30,31]. The picture library seemed to strengthen the mothers’ ability to report the correct food and reduce misunderstandings. However, the pilot study revealed many desired additions to both the picture library and booklet.

Portion size estimation is one of the main challenges in dietary assessment studies. Estimating amounts eaten other than direct weighing may contribute to a source of error, both among children and adults [7,22,24,32]. The photographic booklet was considered to be a good tool for estimating portion sizes among the field workers and the mothers, as has also been reported in several other studies [21,24,30,33]. A study by Lillegaard *et al.* (2005) showed that children and adolescents could accurately estimate portion sizes of pre-weighed foods by viewing photographs, approximately 60% of the comparisons were made correctly [24]. The estimations were more accurate when the served portions had the exact appearance as the food portrayed in the photographic booklet [24]. Thus, the arguments can be made that more picture series in our photographic booklet may be favorable rather than using pictures of similar foods. The studies further emphasize the importance of validation studies to test the applicability of photographs for estimating current portions and actual consumption [21,22], especially among immigrant groups [13]. This was not done in this pilot study, but should be considered in the future.

Assessing children’s food intake accurately can be difficult for a number of reasons. Infants and toddlers cannot account for their food intake, but parents are seen as reliable sources when affirming their children’s consumption of food [25,34]. Efforts should be made to assess foods eaten outside the home or with other caretakers; for instance, at the kindergarten or with family members. A possible challenge may be that the level of reporting and motivation may vary for each caretaker [7]. In the pilot, one of the Somali fathers was on paternity leave and was in charge of the child’s diet at the time; therefore, he was interviewed together with the mother. Among the Iraqi mothers, only one mother reported that her child had spent much of the day with a nanny. Although, this did not apply for many of the mothers in the pilot, it should be taken into consideration for larger studies and dietary assessment of somewhat older children. Potential solutions may be to ask the mothers prior to the interview if the child has other caretakers and if it may be possible to include them to obtain information about their child’s food consumption during their supervision.

In regard to EEI, it seemed to correspond well with the EER for most of the children (within ± 2 SD of the average of the two estimates) except for three. The comparison of EEI and EER has some weaknesses and can only give an indication of whether the method is suitable for capturing habitual energy intake on a group basis. First, each child’s EER might not reflect the true energy requirement of the child because an energy requirement is highly variable between children of the same age and weight [35]. There is also intraindividual variation in energy requirement for children, depending on their physical activity level and growth rate [35]. Second, the energy intake measure was simply averaged over the two days without adjustment for intraindividual variation over time. Thus, it may not be representative of habitual energy intake [36]. Although the sample size was small, it was encouraging that there was no consistent over- or underreporting of EEI compared to EER.

Recruitment of study participants in itself was challenging and time consuming in this pilot study, as it was difficult to come in contact with the target group. This was mostly due to wrong contact numbers registered on several mothers when tried to reach by phone. Some reasons for refusals were that they were not interested, skeptical, or had to consult their partner. It was necessary to seek the mothers through several methods and many did not show up to appointed interviews. The use of bilingual field workers was an advantage and enabled the recruitment of mothers who did not speak Norwegian. Challenges related to recruitment when conducting dietary studies with immigrants have previously been reported [6,26]. Most studies conducted with a European immigrant population group have also used nonprobability sampling methods, such as the convenience sampling method [6]. The need for extra effort in recruiting participants has been described, such as using bilingual field workers, involving key leaders and including places of worship and media, to overcome cultural barriers and ensure representativeness [6]. Although the convenience sampling method may lead to the inclusion of highly motivated participants, there seemed to be variations in the background characteristics of the mothers included in the pilot.

Based on the pilot study presented, some suggestions were made for improving the 24-h multiple-pass recall method. Observations of the interviews showed that the field workers were not actively using the protocol, and a possible solution is to incorporate the protocol into the registration form. Other important suggestion were to include more pictures in the library and supply the photographic booklet with portion sizes of bread in particular, but also of foods such as lasagna, pancakes and other portion sizes of meat, fish, fruits and vegetables. Furthermore, a more thorough training and follow-up of the field workers would be required to increase the quality of the data collection.

## 5. Conclusions

Experiences from the current study indicate that the 24-h multiple-pass recall method with inclusion of visual tools is appropriate method for assessing dietary intake among toddlers of Somali- and Iraqi-born mothers living in Norway. The picture library and photographic booklet were considered to have an added value to the method to aid to identify and describe foods and beverages consumed. However, for the method to be applicable, there is a need for thorough training and follow-up of the field workers during data collection and an update of the picture library and photographic booklet to capture foods, which were not included.
